# Presence of Teen Clubs and its Association with Clinic Attendance among Adolescents Living with HIV in Tanzania: A Retrospective Cohort Study

**DOI:** 10.24248/eahrj.v7i2.735

**Published:** 2023-11-30

**Authors:** Ephrasia Hugho, Theopista Masenga, Jenny Renju, Ola Johanpour, Gretchen Antelman, Sajida Kimambo, Frederick Haraka, Sia E. Msuya

**Affiliations:** a Department of Epidemiology and Biostatistics, Institute of Public Health, Kilimanjaro Christian Medical University College, Moshi, Tanzania; b Biotechnology Research Laboratory, Kilimanjaro Clinical Research Institute, Moshi, Tanzania; c Elizabeth Glaser Pediatric AIDS Foundation, Dar es Salaam, Tanzania; d Department of Community Health, Kilimanjaro Christian Medical Centre, Moshi, Tanzania

## Abstract

**Introduction:**

Adolescent's living with Human Immunodeficiency Virus (HIV) are widely reported to have poor adherence to antiretroviral therapy (ART) due to stigma and fear of discrimination. A “Teen club” is an adolescent centered intervention designed to reduce social stigma and improve adherence and retention in HIV care. In this study we determined how the availability of teen clubs within routine HIV care and treatment facilities affects adolescents' clinic attendance.

**Methods:**

We conducted a retrospective cohort study using secondary data from routine clinic records on adolescents (10–19 years) who started ART between 2010 and 2016, and had documented clinic visits between March 2017 and September 2017 at HIV care and treatment clinics (CTC) in northern and central Tanzania. Good clinic attendance was defined as attending at least four monthly clinic visits during the 7-month follow-up period. A Poisson model with robust standard errors was used to assess the relationship between presence of a teen club at health facilities and good clinic attendance adjusting for other factors including sex, age at ART initiation, duration on ART, WHO clinical stage and health facility level.

**Results:**

Of the 2839 adolescents, 73.1% had good clinic attendance. Good clinic attendance was independently associated with availability of a teen club at the health facility (aRR=1.16, 95%CI:1.09–1.21) and been on ART for >2 years (aRR=1.15, 95%C1.02–1.30). Adolescents aged 15–19 years during this study were less likely to have good clinic attendance (aRR= 0.93, 95%CI:0.88–0.98) than those aged <15 years. Sex and WHO clinical stage were not associated with good clinic attendance.

**Conclusion:**

Teen clubs improved visit adherence among adolescents in HIV care and treatment. Further qualitative research should be conducted to explore adolescents' perception of teen clubs as well as other enablers to clinic attendance.

## INTRODUCTION

In 2016 in Tanzania, antiretroviral therapy (ART) coverage was 48% among children (0–14 years) and 63% among adults.^1^ Despite the rapid scale up of ART, adolescents living with HIV (ALHIV) face challenges in accessing and remaining engaged with HIV care and treatment.^2,3^ Poor adherence to scheduled visits has been associated with virological failure and drug resistance.^4^ Age,^5–7^ stigma and fear of discrimination; poverty and inadequate economic resources; education and HIV/ART literacy^8–9^ are individual factors that affect care retention and health outcomes. Various inter-relational factors are also influential, such as social participation, support structures, culture and religion.^3,10^ Finally, structural barriers to accessing the health facility include distance or transportation costs; operating hours, permissions to leave school, and waiting times.^7^

In Malawi, teen clubs met at a designated time and on the same day adolescents were also able to receive all their HIV related care.^11^ In Uganda, Family Clinic Days (FCD), whereby children, adolescents and their family members are given priority care, achieved 75–100% attendance.^12^ Similarly, Kenya improved clinic attendance through their Youth and Adolescent Friendly Services program in 2013.^13^

In 2016, the Ministry of Health in collaboration with Elizabeth Glaser AIDS Foundation (EGPAF) implemented the 53 Teen clubs in northern and central Tanzania to provide psychosocial support to ALHIV and are led by adolescent peer ambassadors who have extensive knowledge on HIV and stigma. The psychosocial support clubs are intended to help adolescents deal with emotional challenges they face from traumatic experiences such as not having access to basic necessities of life. Through this, teen clubs provide adolescents with a safe and supportive space to discuss sexual and reproductive health issues with their peers. By creating a sense of community and shared experience, teen clubs can reduce stigma and discrimination around health issues and provide adolescents with a supportive network of peers who may encourage them to seek out clinical services. They provide avenue for the people to come together, interact and learn skills that will enable them to make informed decisions when they face challenges in life. The EGPAF teen club model has education sessions which provide correct information on sexual and reproductive health, peer psychosocial support, positive change in mind and attitude towards prevention of Sexually Transmitted Infections (STIs) through provision of adolescent friendly services and improving adherence and retention to care among HIV positive adolescents. It also imparts adolescents with life skills for managing both physical and social developmental changes occurring at their age.

Teen club activities were scheduled on Saturdays, and in order to become a member of the club, individuals must meet the following criteria: aged between 10 and 19 years, be aware of their HIV status, and be receiving HIV care services at the specific site. In this study, we compared HIV clinic attendance amongst adolescents receiving HIV care in health facilities with teen clubs to those in facilities without teen clubs.

## METHODS

### Study Design and Setting

We conducted a retrospective cohort study to assess the association between teen club presence and clinic attendance in a population of ALHIV attending ART care from March to September 2017. The study was conducted in 144 health facilities that offer CTC services. The facilities included in the analysis were located in four regions: Arusha (42 health facilities), Dodoma (36 health facilities), Kilimanjaro (44 health facilities), and Tabora (77 health facilities). As of March 2016, teen clubs were running in 51 health facilities; 16 in Arusha region, 13 in Kilimanjaro region, 3 in Dodoma region and 19 in Tabora region.

### Eligibility Criteria

This study included all ALHIV aged 10–19 years who started ART between 2010 and 2016, who were registered and accessing ART care in the selected health facilities and had at least one documented clinic visit between 1^st^ March and 30^th^ September, 2017. All CTC sites with at least 10 ALHIV registered in ART care were eligible for the study.

### Sample Size and Sampling

**FIGURE 1:**
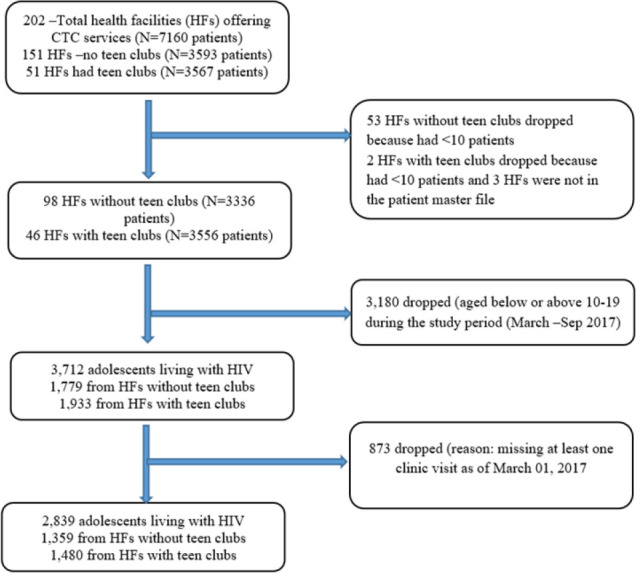
Sites and Participants Selection Process

This was a secondary analysis of the available CTC data. All eligible adolescents from 144 CTC sites were included in the study. A total of 2839 participants were obtained for analysis. The Power of the study was calculated by using formula for power calculation for two proportions *(power two proportions p1 p2, n1( ) n2( ).* With 1359 adolescents in the unexposed group (from health facilities without teen clubs) and 1480 in exposed group (from health facilities with teen clubs), the analysis had 100% power to detect 10% difference in the proportion of adolescents who had optimal clinic attendance in the two groups at 5% significant level. Figure 1 shows the sites and participant selection process.

### Data Source and Study Variables

Data were extracted from routine HIV care and treatment patient records. The primary exposure was availability of the teen club at the health facility. Other variables included sex of the patient, age during the study period, age at ART initiation, reported ARV adherence, duration on ART (time since the initiation of ARVs), World Health Organization (WHO) HIV clinical stage and health facility level (dispensary, health center or hospital).

Good clinic attendance was the primary outcome, defined as having attended at least four visits during the study period, where according to the Tanzanian standard of care for ALHIV, monthly clinic visits were recommended.^13^ The teen clubs were offered monthly and provided clinical services and ART refills. Other outcomes observed at the end of the study follow-up were loss to follow-up (LTFU) defined as not having attended the clinic by the end of the study follow-up period for >90 days ^14^, death or stopping ART, and retention (not LTFU, died or stopped ART).

### Data Analysis

Data analysis was performed using STATA version 15. Descriptive statistics (measures of central tendency, frequencies, and percent) were used to summarize continuous and categorical variables. In both the univariate and multivariable analyses, we used modified Poisson regression with robust error variances^19^ to obtain risk ratios (RR) and precise confidence intervals for the association between exposure variables and good clinic attendance. A complete case analysis was performed and only variables with a *P* value of less than 0.2 in the univariate analysis were included in the multivariable model. Statistical inference for significant association was made at 95% confidence interval and Wald test *P* value <0.05. The best model fit was the one with the smallest Akaike Information Criterion (AIC) value.

### Ethical Considerations

Ethical approval for this secondary analysis was obtained from the Research and Ethics Committee of the Kilimanjaro Christian Medical University College-Tumaini University Makumira (No. 2032). This study was also conducted under the protocol *Reaching 90-90-90 evaluation of patient and program characteristics associated with identification of adults and children living with HIV, ART initiation and retention in Tanzania*, describing secondary analyses objectives for analyzing the National HIV Care and Treatment data from facilities supported by the EGPAF; approved by the National Research Ethics Committee under the National Institute for Medical Research (NIMR/HQ/R.8a/Vol IX/2185).

## RESULTS

### Characteristics of Study Participants

A total of 2839 adolescents from 144 health facilities were included in our analyses. The mean (SD) age of the participants was 14.4(2.3) years. The majority (91.4%) of the participants started ART at the age of ≤15 years and 77.7% had been using ART for more than 2 years.

Clinician reported ARV adherence was charted as “good” at every visit for 85.4% of the participants. Participant characteristics were significantly different in terms of health facility level, duration on ART and region of residence when comparing teen club sites versus no teen club sites (Table 1).

**TABLE 1:** Characteristics of the Study Participants by Availability of Teen Clubs (N=2839)

Variable	Total		Health facility status				P Value
			No Teen club (n=1359)		Teen club (n=1480)		
	n	%	n	%	n	%	
Sex							
Male	1265	44.6	600	44.1	655	44.9	.675
Female	1574	55.4	756	55.9	825	55.1	
Age at ART initiation							
<10 years	1230	43.3	574	42.2	656	44.3	.185
10–14	1366	48.1	656	48.3	710	48.0	
15–19	243	8.6	129	9.5	114	7.7	
Age during the study							
<15	1935	68.2	915	67.3	1020	68.9	.364
15–19	904	31.8	444	32.7	460	31.9	
Baseline WHO Stage							
1	519	18.3	253	18.6	266	18.3	.072
2	946	33.3	477	35.1	469	33.3	
3	1131	39.8	516	38.0	615	39.8	
4	240	8.6	110	8.1	130	8.6	
Missing	3	0.1	3	0.2	0	0	
Reported ARV Adherence							
Good	2424	85.4	1154	84.9	1270	85.4	.138
Poor	126	4.4	71	5.2	55	4.4	
Missing	289	10.2	134	9.9	155	10.2	
Health facility level							
Dispensary	274	9.7	212	15.6	62	4.2	<.001
Health center	932	32.8	508	37.4	424	28.6	
Hospital	1633	57.5	639	47.0	994	67.2	
Region							
Arusha	647	22.8	110	8.1	537	36.3	<.001
Dodoma	613	21.6	454	33.4	159	10.7	
Kilimanjaro	864	30.4	476	35.0	388	26.2	
Tabora	715	25.2	319	23.5	396	26.8	

### Good Clinic Attendance and its Associated Factors

Overall good clinical attendance was 73.1%. A significantly higher proportion (78.2%) of ALHIV who attended facilities with teen clubs completed the good number of clinic visits during the study period compared to adolescents attending clinics without teen clubs (67.6%; *P* value < .001). Other outcomes observed during the study period include retention care (92%) and loss to follow up (7.5%) Table 2.

**TABLE 2:** Outcomes During Follow up Period (N=2839)

Outcomes	Total		No teen club (n=1359		Teen club (n=1480)	
	n	%	n	%	n	%
Good clinic attendance	2075	73.1	918	67.6	1157	78.2
Retained	2611	92.0	1226	90.2	1385	93.6
LTFU	212	7.5	129	9.5	83	5.6
Stopped ART	4	0.1	2	0.2	2	0.1
Died	13	0.4	2	0.2	10	0.7

In the adjusted analysis, good clinic attendance was independently associated with having a teen club at the health facility, age and duration on ART (Table 3). We observed a 15% higher odds of achieving good clinic attendance among adolescents attending facilities with a teen club compared to those without a teen club (adjusted RR: aRR=1.15, 95% CI:1.09–1.20). Compared to adolescents aged <15 years, those aged 15–19 years were 7% less likely to have good clinic attendance (aRR=0.93, 95% CI: 0.88–0.98). Adolescents who had been on ART for more than 2 years had 15% good clinic attendance compared to those on ART for less than two years (aRR=1.15, 95% C1.02–1.30).

**TABLE 3:** Factors Associated with Good Clinic Attendance

Variable			Univariate analysis		Multivariable analysis	
	n	n (%)	cRR (95% CI)	P-value	aRR (95% CI)	P value
Health facility status						
No teen club	1359	918 (67.6)	1.00		1.00	
Teen club	1480	1157 (78.2)	1.16(1.10 – 1.21)	<.001	1.15 (1.09 – 1.20)	<.001
Sex						
Male	1265	938 (74.2) 1.00	1.00			
Female	1574	1137 (72.2)	0.97 (0.93 – 1.01)	.251	0.94 (0.93 – 1.02)	.364
Age						
<15	1935	1450 (74.9)	1.00		1.00	
15–19	904	625 (69.1)	0.92 (0.88 – 0.97)	.002	0.93 (0.88 – 0.98)	.003
Baseline WHO Stage						
1	519	399 (76.9)	1.00		1.00	
2	946	661 (69.9)	0.91 (0.85 – 0.97)	.003	0.90 (0.84 – 0.96)	.001
3	1131	830 (73.4)	0.95 (0.90 – 1.01)	.121	0.93 (0.88 – 0.98)	.014
4	240	182 (75.8)	0.99 (0.91 – 1.07)	.754	0.96 (0.88 – 1.04)	.315
Unknown	3	3 (100)	1.30(1.24 – 1.36)	<.001	1.58(1.46 – 1.72)	<.001
Duration on ART						
<1 year	138	90 (65.2)	1.00		1.00	
1–2 years	494	322 (65.2)	0.99 (0.87 – 1.15)	.994	1.00 (0.86 – 1.15)	.952
More than 2 years	2207	1663 (75.4)	1.16(1.02 – 1.30)	.023	1.15 (1.02 – 1.30)	.025
Health facility level						
Dispensary	274	196 (71.5)	1.00	–	–	–
Health center	932	675 (72.4)	1.01 (0.93 – 1.10)	.774	–	–
Hospital	1633	1204 (73.1)	1.03 (0.95 – 1.12)	.459	–	–

## DISCUSSION

The findings of this study suggest that the availability of teen clubs within the health facility could improve adolescents' monthly attendance at the ART clinic. Interestingly, adolescent retention into ART care was good for both study groups (with and without teen clubs). Further, younger ALHIV had a higher odds of having a good clinic attendance during the study period.

Over three-quarters of adolescents receiving ART at health facilities with teen clubs had good clinic attendance.

Teen clubs provide adolescents with time to share life experience with their peers^16^ thus providing strong peer support. Also, adolescents attending facilities with teen clubs might have been motivated to attend due to the availability of sexual and reproductive health education sessions, or to benefit from the integration of psychosocial/peer support and HIV clinical services provided during the teen club meetings.^17^ The teen clubs are offered on a Saturday, they do not interfere with school hours^7^ and may provide a more private environment for adolescents.^18^ Further, good clinical attendance might have been influenced by awareness and acceptance of their disease status (disclosure) which is criterion for joining the teen clubs perhaps increasing the adolescents' understanding of the importance of being on long time ART.^5^ Similar findings were reported in Malawi and Uganda where adolescent support groups were found to reduce rates of loss to follow up and improve clinic attendance.^10–11^

Older adolescents (15–19 years old) were found to be less likely to have good clinic attendance compared to younger adolescents. Poor clinic attendance at this age might be due to other family and school responsibilities.^7^ Also older adolescents may be negotiating sexual relations and facing challenges disclosing their status,^5^ which could lead them to discontinue their care. A fear of needing to transition to adult care has also been shown to repel older ALHIV from attending clinics.^6^ Similar findings were observed in Uganda and Malawi where it was reported that older adolescents had higher risk of not remaining in care.^10, 19^ There is a need for providers and teen club facilitators to implement age-appropriate strategies to support adherence to clinic appointments, recognizing the increasing need for autonomy and self-management among older adolescents, in order to maintain access to HIV care.

Adolescents who were on ART for more than two years were found to have good clinic attendance compared to those on ART for less than two years. Good clinic attendance leading to better retention in care among patients on ART for long time has also been reported in the United States.^9^ Perhaps those on ART for a longer period are knowledgeable about medication adherence and they believe that medication enables them to live longer.^8^ Having no problems in taking medications (little or no side effects) also plays an important role in retaining patients in clinics hence maintaining their practice of attending clinic for medication refill.^7^

### Limitations

Teen clubs were not evenly distributed across the study areas since the priority for teen club establishment was given to the high level health facilities with high volume of patients.

Participants receiving ART care in health facilities without teen clubs differed in some characteristics from those in teen club site thus might have affected our results. Further, this study was not able to assess other factors such as distance to health facility or other aspects of quality of care, including adolescent awareness and attendance of the clubs.

## CONCLUSION

The findings of this study indicate that adolescents enrolled into teen clubs are more likely to have good clinic attendance during the study period, supporting growing evidence that teen clubs can support adolescents to achieve good clinic attendance. There is a need for newly enrolled adolescents in HIV care and treatment to join the support group and assess whether frequency and consistency of adolescent participation in the teen club relates to improved health outcomes. Qualitative research should be conducted to explore adolescents' perception of teen club and other ART outcomes as well as other enablers of clinic attendance.
